# Laryngeal Carcinoma in a Pediatric Patient - A Case Report

**Published:** 2019-07

**Authors:** Santosh-Kumar Swain, Mahesh-Chandra Sahu

**Affiliations:** 1 *Department of Otorhinolaryngology, IMS and SUM Hospital, Siksha“O”Anusandhan University (Deemed to be), K8, Kalinganagar, Bhubaneswar-751003, Odisha, India.*; 2 *Directorate of Medical Research, IMS and SUM Hospital, Siksha“O” Anusandhan University (Deemed to be), K8, Kalinganagar, Bhubaneswar-751003, Odisha, India.*

**Keywords:** Laryngeal carcinoma, Pediatric patient, Squamous cell carcinoma

## Abstract

**Introduction:**

Carcinoma of the larynx is an extremely uncommon clinical entity in pediatric age. The diagnosis of the laryngeal carcinoma is often delayed due to the low index of suspicion. The factors contributing to delayed diagnosis include the similarity of its symptoms to common benign lesions of the larynx in childhood and difficult examination of the larynx in pediatric patients. The treatment of laryngeal carcinoma is still a challenge due to the lack of available guidelines among pediatric patients.

**Case Report:**

An 11-year-old male presented with hoarseness of voice over the last 3 month and was diagnosed with laryngeal carcinoma based on the fiberoptic nasopharyngolaryngoscopy examinations and biopsy. He was treated with a complete course of radiotherapy.

**Conclusion:**

This case is reported due to the paucity of the laryngeal carcinoma cases among pediatric patients in medical literature. The obtained results will create awareness among clinician to rule out laryngeal carcinoma in pediatric patients with laryngeal symptoms, such as the hoarseness of voice which will help early diagnosis and treatment.

## Introduction

Laryngeal malignancy is the most well-known lesion of the upper aerodigestive tract in elderly age group. The majority of the patients have a history of smoking and alcohol utilization. In addition to human papillomavirus infection (strain 16,8,33), other hazardous risk factors that are associated with occupation and hereditary variables include wood dust, fuel vapor, and diesel (1). The laryngeal carcinoma in children is greatly uncommon and its manifestations are nonspecifically leading to postponement in findings. The frequency and commonness of laryngeal carcinoma are not known in the pediatric age group. Past history of irradiation and intermittent laryngeal papillomatosis are considered as real hazard factors for laryngeal carcinoma in youngsters (2). The laryngeal carcinoma in children are frequently not anticipated in differential analysis with the roughness of voice, dysphagia as well as dynamic upper airway route block in pediatric age; therefore, the diagnosis is made moderately late. Here, we present an instance of laryngeal carcinoma with its clinical features, investigations, and treatment, in a pediatric patient.

## Case Report

**Fig 1:**
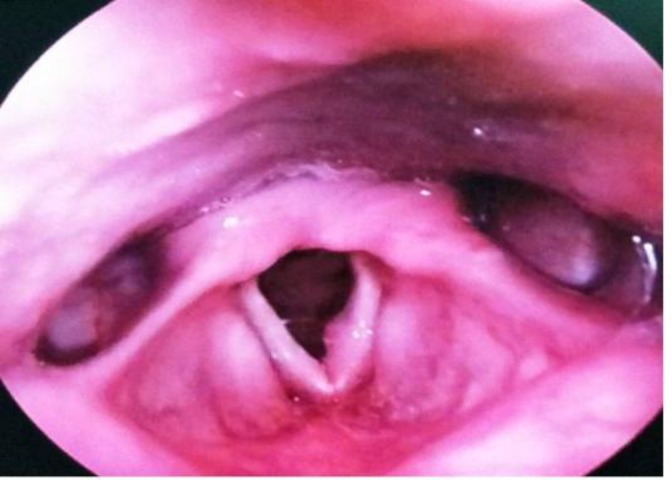
Fiberoptic Nasopharyngolaryngoscopy showing irregular growth at anterior part of the left vocal cord

**Fig 2:**
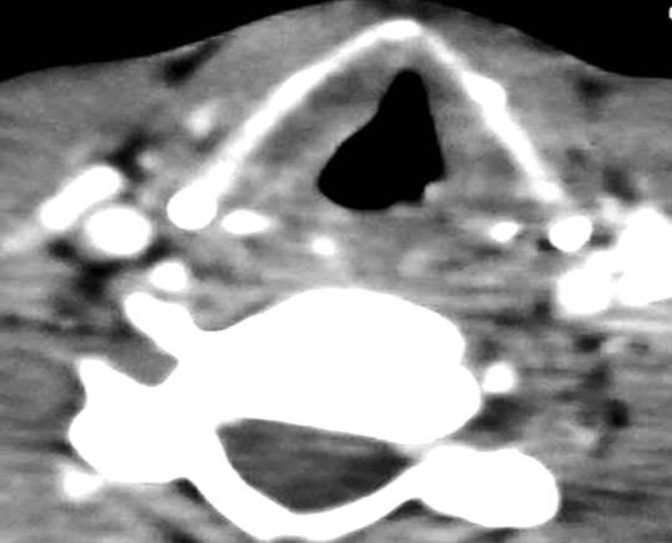
CT scan picture of the glottic growth at left vocal cord

An 11-year-old male referred to the outpatient Department of Otorhinolaryngology with a complaint of throat irritation and hoarseness of voice over the past 3 months. He had no history of breathing difficulty, dysphagia, upper respiratory tract contamination, voice misuse, tobacco use, and previous radiation presentation. Moreover, there was no family history of head and neck malignancy or any hereditary variation from the norm related to the improvement of laryngeal carcinoma in youth.His past medical history was unremarkable. Indirect laryngoscopy revealed a growth in the left vocal cord. Fiberoptic nasopharyngolaryngoscopy showed an irregular growth in the left vocal cord with impaired cord mobility (Fig.1). There was no supraglottic and subglottic extension of the growth and swollen lymph node in neck. Computed tomography (CT) scan and magnetic resonance imaging (MRI) of the neck showed enhancement in the left vocal cord; moreover, other parts of the neck were within normal limit (Fig.2).

**Fig 3:**
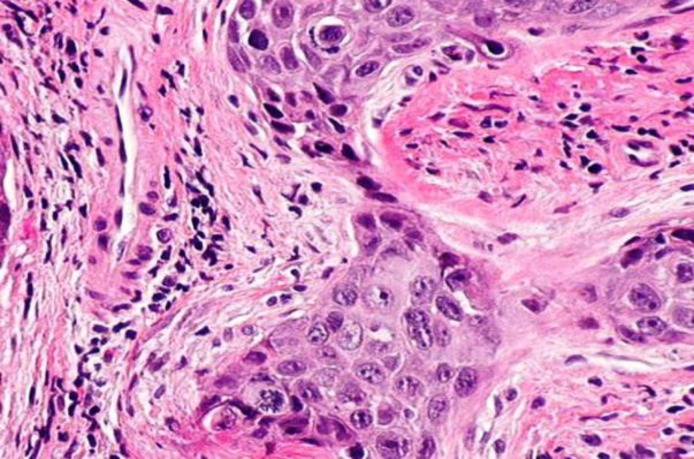
Photomicrography showing histopathological picture of the squamous cell carcinoma(H& E stain,400X).

Furthermore, no invasion or extralaryngeal spread to laryngeal cartilages was observed in this case report. In addition, thoracic CT scan was not indicative of distant metastasis. A biopsy was done under general anesthesia and histopathological examination showed moderately differentiated non-keratinizing squamous cell carcinoma (Fig.3). 

**Fig.4:**
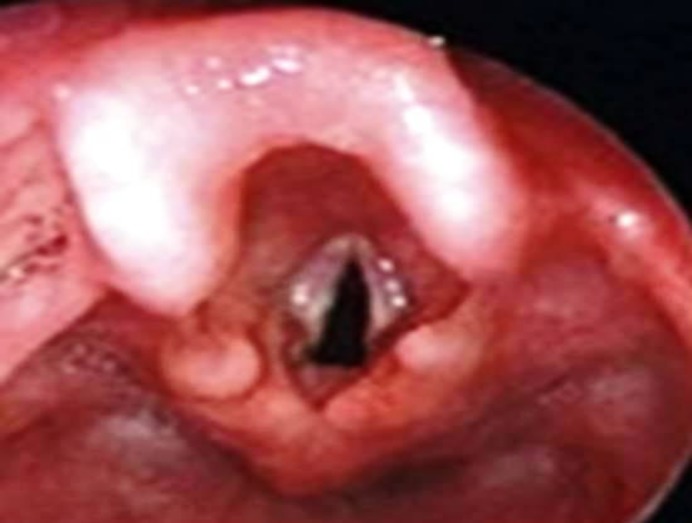
Post radiotherapy picture of Larynx showing no evidence of malignant lesion

It was inferred that the child had stage 1 glottic cancer (i.e., T1N0M0). Given the early stage of this disease, the patient was subjected to radiotherapy. After the fulfillment of radiotherapy, larynx was again inspected with no proof of lesion after 3 months (Fig.4). The patient did not require tracheostomy due to the treatment of laryngeal carcinoma and satisfactory laryngotracheal airway.

## Discussion

The laryngeal carcinoma is an extremely rare clinical entity in children. The first case of pediatric laryngeal carcinoma was reported in 1868 (3). The head and neck malignancies represent 2 to 10% of all the tumors (solid tumors) of pediatric ages. Moreover, laryngeal carcinoma accounts for under 0.1% of all the head and neck malignancies in childhood (4). Most tumors of the larynx in pediatric patients include carcinoma and rhabdomyosarcoma (5). Other malignant tumors of the larynx are minor salivary gland carcinoma, primitive neuroectodermal tumor, and metastatic lesions. The majority of laryngeal carcinoma is squamous cell carcinoma and other types include adenocarcinoma and mucoepidermoid carcinoma of the minor salivary glands (3). The laryngeal carcinoma is usually confined to the vocal cords and less commonly observed in supraglottis and subglottis.

There is controversy regarding risk factors predisposing to pediatric laryngeal carcinoma. However, the important predisposing factor for laryngeal carcinoma in children is radiation therapy for benign head and neck lesions, such as juvenile laryngeal papillomatosis. Nevertheless, carcinomatous lesions may be associated with juvenile papillomatosis without prior irradiation (6). 

Biopsy and histopathological examination are essential to rule out malignancy of the larynx in children. Other known risk factors for laryngeal carcinoma are active and passive smoking and exposure to chemicals, such as asbestos. Laryngeal infection with human papillomavirus (types 18 and 13) have been associated with laryngeal carcinoma in the pediatric age group (7).

Chromosomal abnormalities are documented in one study in which chromosomal translocation (15;19) was observed in a pediatric supraglottic carcinoma (8).

Tobacco use is often considered as a risk factor for laryngeal cancer in adults, whereas few cases are detected in pediatric age due to tobacco consumption (9). The risk of laryngeal carcinoma increases with age and the mortality rate of laryngeal carcinoma in pediatric age is approximately 38% (5).

 The clinical presentations of the pediatric patients suffering from laryngeal carcinoma include dysphonia, stridor, dysphagia, foreign body sensation in the throat, and respiratory distress.

These clinical findings may be confused with benign or inflammatory diseases of the larynx in children. In older children, dysphonia may be confused with puberty voice change. All children presenting with dysphonia should be examined with indirect or direct laryngoscopy. If laryngeal symptoms progress or persist in the pediatric age group, a high index of suspicion is required to rule out laryngeal carcinoma where appropriate imaging and biopsy are warranted. The differential diagnoses of laryngeal carcinoma in the pediatric age group are papillomas, rhabdomyosarcoma, subglottic hemangioma, and adenocarcinoma of the minor salivary gland.

Due to vocal cord changes during puberty, recurrent upper respiratory tract infection, or voice abuse, there is often delay in the diagnosis of laryngeal carcinoma in pediatric patients. The majority of the pediatric patients present with an extensive lesion at the time of diagnosis. The delayed diagnosis is also attributed to difficult visualization of the larynx using laryngeal mirror examination in children. The larynxes of pediatric patients are adequately assessed by trans nasal flexible laryngoscopy. A high index of suspicion is often essential to diagnose the laryngeal carcinoma in pediatric patients. The definitive diagnoses of laryngeal lesions are performed by endoscopic findings and biopsy with histopathological examination.

Due to the rarity of laryngeal carcinoma in childhood, there is no standard rule for the treatment of this disease; moreover, there remains no consensus as to the most relevant therapy (10). Treatment of laryngeal carcinoma in pediatric patients presents numerous issues. It is usually difficult to inform the youthful patients, their relatives, or parents, about the concept of the disease, treatment decisions, and its delayed consequences. 

Precise and early-stage diagnosis is critical for the management of pediatric laryngeal carcinoma. Reviews of clinical cases reported in the literature are not good enough to use for making treatment decisions due to the lack of sufficient information regarding staging, management, and outcome.

 The laryngeal carcinoma is commonly treated with either surgery or radiation therapy (RT); however, there are patients who receive both treatments. This disease is less commonly treated through chemotherapy. In pediatric patients, radical radiotherapy can cause significant growth retardation of both bone and soft tissue leading to deformity and dysfunction. Delayed complications of radiotherapy in children are facial growth retardation, neuroendocrine dysfunction, dental abnormalities, visual problems, and hypothyroidism. Late complications resulted from radiotherapy after the age of 10 years includes chondronecrosis, esophageal atresia, second malignancy, and brain hemorrhage (11). In the early stage of the lesion, surgery may be useful in order to withhold the radiation in the pediatric age group. Laser cordectomy is often beneficial in early-stage glottic carcinoma (12). Surgical treatment is not often satisfactory for the advanced laryngeal carcinoma (i.e., stage 3 or 4) in children. In addition, the complication of the treated lesions may also lead to morbidity (13). In one study, out of 17 children with laryngeal carcinomas, 13 cases had glottic lesions and 4 patients suffered from supraglottic lesions. With regard to the treatment of 13 patients, glottic carcinoma, cord stripping, partial laryngectomy, total laryngectomy, radiotherapy, laryngectomy with RT, and chemotherapy in addition to RT was performed in 2,3,2,3,1, and 2 cases, respectively (3,14,15).

Although pediatric laryngeal carcinoma is extremely uncommon, the clinician ought to be aware of this disease due to the differential occurrence of laryngeal lesions in pediatric patients. The patients undergo chemotherapy, radiation therapy alone or combined with a medical procedure the time immediately after diagnosis or at the late stages of the disease. Moreover, these patients need follow-ups in their whole duration of life to check laryngeal carcinoma and probable incidence of malignancies.

## Conclusion

Laryngeal carcinoma is extremely rare in the pediatric age group. There is often delay in diagnosis because clinical symptoms may be misinterpreted as non-malignant lesions of the larynx. The predisposing factors causing laryngeal carcinoma is still unclear. The laryngeal carcinoma in pediatric patients is more aggressive than that among adult. This may be due to delayed diagnosis in children with the late stage of the disease. The treatment is often individualized and aimed at personalized outcomes. The presented case in this case report will raise awareness of clinicians regarding the laryngeal carcinoma in pediatric patients which leads to early diagnosis and effective treatment.
